# Predicting visual recovery in pituitary adenoma patients post-endoscopic endonasal transsphenoidal surgery: Harnessing delta-radiomics of the optic chiasm from MRI

**DOI:** 10.1007/s00330-023-09963-9

**Published:** 2023-07-24

**Authors:** Yang Zhang, Junkai Zheng, Zhouyang Huang, Yuen Teng, Chaoyue Chen, Jianguo Xu

**Affiliations:** 1 Department of Neurosurgery, West China Hospital, Sichuan University, No. 37, GuoXue Alley, Chengdu, 610041 China; 2 Department of Radiology, West China Hospital, Sichuan University, No. 37, GuoXue Alley, Chengdu, 610041 China

**Keywords:** Pituitary adenoma, Magnetic resonance imaging, Optic chiasm, Machine learning

## Abstract

**Objectives:**

To investigate whether morphological changes after surgery and delta-radiomics of the optic chiasm obtained from routine MRI could help predict postoperative visual recovery of pituitary adenoma patients.

**Methods:**

A total of 130 pituitary adenoma patients were retrospectively enrolled and divided into the recovery group (*n* = 87) and non-recovery group (*n* = 43) according to visual outcome 1 year after endoscopic endonasal transsphenoidal surgery. Morphological parameters of the optic chiasm were measured preoperatively and postoperatively, including chiasmal thickness, deformed angle, and suprasellar extension. Delta-radiomics of the optic chiasm were calculated based on features extracted from preoperative and postoperative coronal T2-weighted images, followed by machine learning modeling using least absolute shrinkage and selection operator wrapped with support vector machine through fivefold cross-validation in the development set. The delta-radiomic model was independently evaluated in the test set, and compared with the combined model that incorporated delta-radiomics, significant clinical and morphological parameters.

**Results:**

Postoperative morphological changes of the optic chiasm could not significantly be used as predictors for the visual outcome. In contrast, the delta-radiomics model represented good performances in predicting visual recovery, with an AUC of 0.821 in the development set and 0.811 in the independent test set. Moreover, the combined model that incorporated age and delta-radiomics features of the optic chiasm achieved the highest AUC of 0.841 and 0.840 in the development set and independent test set, respectively.

**Conclusions:**

Our proposed machine learning models based on delta-radiomics of the optic chiasm can be used to predict postoperative visual recovery of pituitary adenoma patients.

**Clinical relevance statement:**

Our delta-radiomics-based models from MRI enable accurate visual recovery predictions in pituitary adenoma patients who underwent endoscopic endonasal transsphenoidal surgery, facilitating better clinical decision-making and ultimately improving patient outcomes.

**Key Points:**

*• Prediction of the postoperative visual outcome for pituitary adenoma patients is important but challenging.*

*• Delta-radiomics of the optic chiasm after surgical decompression represented better prognostic performances compared with its morphological changes.*

*• The proposed machine learning models can serve as novel approaches to predict visual recovery for pituitary adenoma patients in clinical practice.*

**Supplementary information:**

The online version contains supplementary material available at 10.1007/s00330-023-09963-9.

## Introduction

Pituitary adenomas are the most prevalent sellar region neoplasm [1, 2]. Patients with pituitary adenoma may suffer from significant visual dysfunctions as the location of tumor growth is in proximity to critical structures for vision, especially referred to as optic chiasm. Compression of the optic chiasm can lead to visual morbidity and subsequent compromised quality of life [3–5]. It is also one of the main indicators for therapeutic intervention [6, 7]. Endoscopic endonasal transsphenoidal surgery (EETS) has become one of the predominant choices for tumor resection, which has shown promising results regarding tumor resection rates and postoperative visual improvement [8].

Identifying prognostic factors of favorable visual outcomes is of major importance, as this can aid clinical decision-making about the benefits of surgical intervention. For patients at high risk for persistent visual impairment, timely initiation of operation and referral for visual rehabilitation after surgery are crucial to preserve visual function and improve quality of life [9, 10]. Previous researchers attempted to identify predictive factors that correlate with the postoperative visual outcome, including tumor size, age, preoperative visual field, and retinal nerve fiber layer (RNFL) thickness [11–14]. Whereas, the results are still controversial as unquantified outcomes, small sample sizes, and partial predictors constitute the limitations of these various types of research. Besides, some parameters they used are either expensive or not easily obtained from routine clinical practice [15–17]. A more practical, convenient method is required to meet the need for individualized patient management strategies.

The noninvasive quantification of medical images is a novel and promising field of research referred to as radiomics. By retrieving high-throughput information from images, radiomics techniques aim at providing personalized medicine approaches by leveraging big-data analytics [18, 19]. With the extraction of thousands of numeric features and careful feature selections, machine-learning classifiers can be designed and trained to answer some crucial questions beyond naked-eyes’ assessment, such as patient outcome prediction [20]. Nowadays, for neurosurgeons and neuroradiologists, who might need most assistance from precise prognostic prediction, the MR scan is still the standard examination for both diagnosis and follow-up of pituitary adenoma patients [21, 22]. Morphological assessment of optic chiasm on MRI is the primary choice to evaluate the compression status of the optic chiasm and visual outcome of patients in clinical work [23, 24]. Moreover, visual MRI evaluation before and after surgery can highlight a series of morphologic recovery of the optic chiasm associated with functional recovery that may be quantified with radiomics features [25]. Considering the visual recovery of pituitary adenoma patients after surgery is a dynamic process, the data involving radiomics feature changes (also referred to as delta-radiomics) of the optic chiasm during surgical decompression would be more informative. Delta-radiomics is a method that analyzes the relative net changes in radiomics parameters using a set of consecutive images to provide a quantitative assessment of the underlying histopathological changes. In recent various types of research, delta-radiomics has been utilized to predict the treatment response and prognosis in many diseases and represents promising performances [26–29]. Therefore, in the current research, with routine MRI, we aimed to investigate if changes in traditional morphological parameters and radiomics features of the optic chiasm after surgical decompression could help predict the visual outcome of pituitary adenoma patients who underwent EETS.

## Materials and methods

### Patient population

**Fig. 1:**
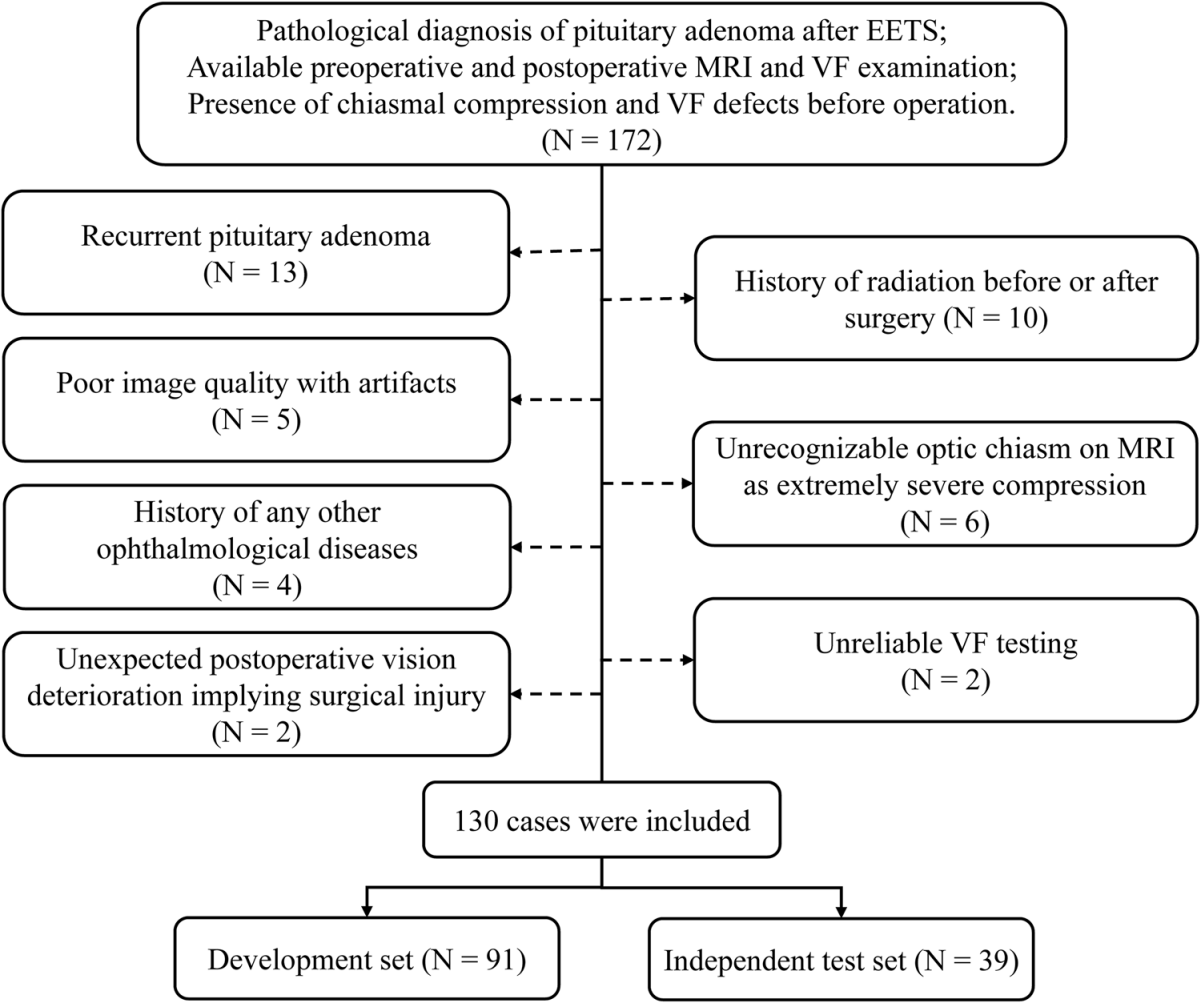
Flow chart of patient enrollment in this study

This retrospective study was approved by the institutional review board, and written informed consent was waived (2021-S-851). The inclusion criteria were patients who (1) underwent EETS in curative intent between March 2018 and January 2021; (2) with pathologically proven pituitary adenoma; (3) presence of tumor-related chiasmal compression and visual field (VF) defects before surgery; (4) with sellar MRI within two weeks preoperatively and within three days postoperatively; (5) with VF testing at 1 year after operation. The exclusion criteria encompassed: (1) recurrent pituitary adenoma (*n* = 13); (2) history of radiation before or after surgery (*n* = 10); (3) poor image quality of MRI with artifacts (*n* = 5); (4) unrecognizable optic chiasm caused by severe tumor compression (*n* = 6); (5) history of any other ophthalmological diseases like cataract or glaucoma (*n* = 4); (6) unreliable VF testing, defined as > 33% false negative responses or false positives responses (*n* = 2); (7) unexpected postoperative VF deterioration (more than a 10 dB decrease in mean deviation) implying surgical injury (*n* = 2) [14]. The flow chart of patient selection was shown in Fig. 1. Furthermore, we collected the clinical characteristics of qualified patients, including age, gender, histological subtype, and Ki-67 index of tumors.

### Ophthalmological assessment

The VF of all qualified patients were assessed with standard automated perimetry (OCTOPUS 900, Haag-Streit Inc; Humphrey Field Analyzer, Carl Zeiss Meditec Inc) preoperatively and postoperatively. A mean deviation (MD) worse than − 3.0 dB was regarded as the VF defect. Patients were divided into two groups according to the results of the VF testing 1 year after surgery: the recovery group (postoperative MD ≥  − 3.0 dB) and the non-recovery group (postoperative MD <  − 3.0 dB), as suggested by previous research [12, 13, 30].

### Image acquisition and morphologic evaluation

The MR images within two weeks before surgery and within three days after surgery were exported for all eligible patients. Considering coronal slices of sellar MRI best demonstrate chiasmal compression and the boundary of the optic chiasm is clearly displayed in T2-weighted imaging (T2WI), coronal T2WI were used for the morphologic evaluation and segmentation of the optic chiasm. The acquisition parameters for T2WI were summarized in Supplementary Materials 1.

**Fig. 2:**
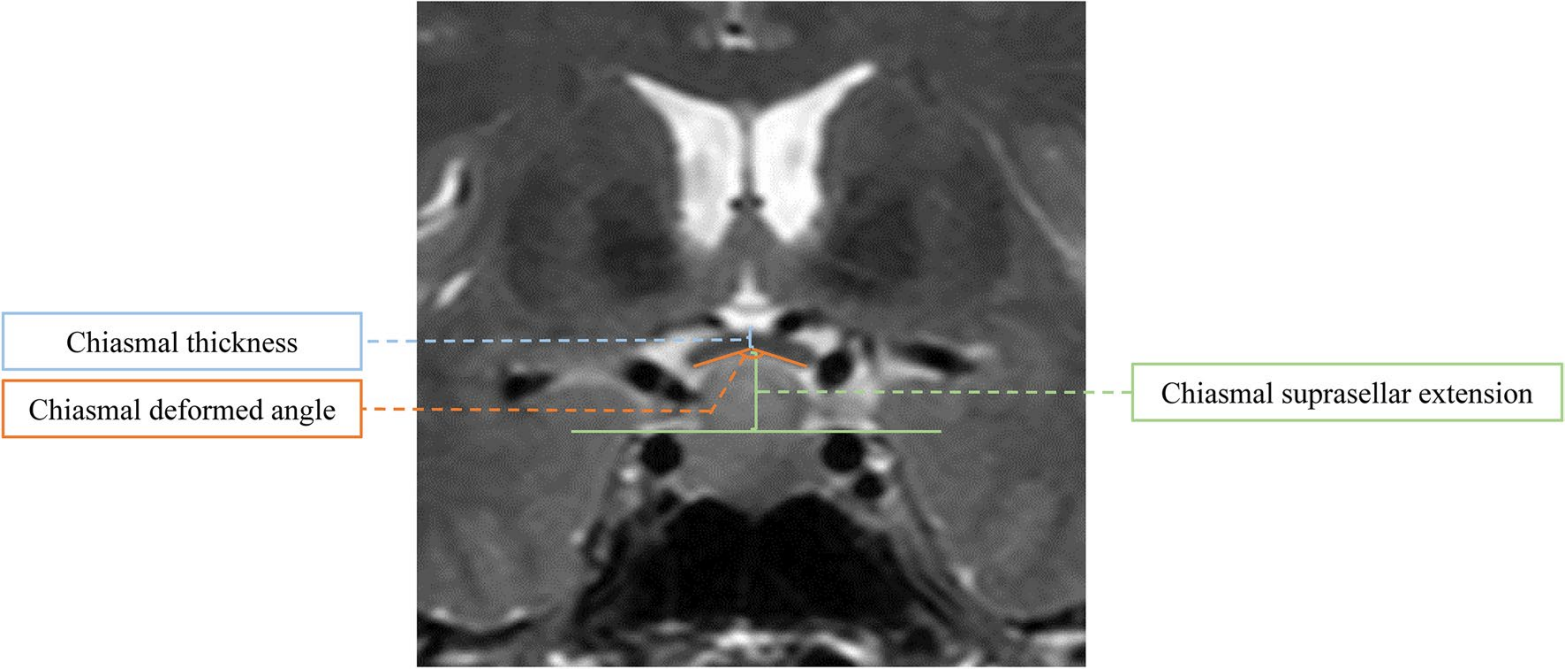
The example of measuring morphological parameters of the optic chiasm on coronal T2-weighted MR images, including chiasmal thickness, chiasmal deformed angle, and chiasmal suprasellar extension

Morphologic parameters of the optic chiasm, including chiasmal thickness, chiasmal deformed angle, and chiasmal suprasellar extension, were measured preoperatively and postoperatively by two neuroradiologists together blinded to patient information (Fig. 2). The chiasmal thickness was measured as the distance between the upper and lower margin of the optic chiasm at the maximal deflection of all slices. The chiasmal deformed angle was defined as the minimal angle created by drawing lines from two sides of the optic chiasm to the midline along the lower margin of all slices. The chiasmal suprasellar extension was defined as the maximal perpendicular distance between the lower margin of the optic chiasm and a reference line at the level of the upper surface of bilateral internal carotid arteries within the cavernous sinus. The absolute change and relative change rate of the above morphologic parameters of the optic chiasm after surgical decompression were also calculated.

### Image preprocessing and radiomics feature extraction

Before image preprocessing, all MR images were anonymized, and all private information and protected health information were blinded from DICOM files. Image preprocessing included image resampling of T2WI to 1 × 1 × 1 mm^3^ voxel resolution and N4ITK bias correction.

Regions of interest (ROI) of optic chiasm were delineated slice by slice by two neuroradiologists separately using the open-source software ITK-SNAP v3.8.0 [31]. The discontinuous portions anterior or posterior to the optic chiasm, regarded as optic nerves or optic tracts, were excluded in ROI delineation. By using the open-source package PyRadiomics v3.0, a total number of 851 quantitative imaging features were retrieved from ROI to decode the radiographic phenotype following the Imaging Biomarker Standardization Initiative (IBSI) guideline [32]. The bin width was set to 5 according to the recommendation of PyRadiomics (https://github.com/AIM-Harvard/pyradiomics). Radiomic features included shape features (*n* = 14), histogram features (*n* = 18), texture features (*n* = 75), and wavelet-based features (*n* = 744). Intraclass correlation coefficients (ICC) of features extracted by two neuroradiologists were calculated to exclude features with significant intra-observer variations. Only radiomics features with ICC > 0.8 in both preoperative and postoperative image sets were considered reproducible and collected for further analysis. The delta-radiomics features depicting the relative change in radiomics features of the optic chiasm obtained from preoperative (Feature_pre_) and postoperative (Feature_post_) images were calculated using the following formula: ΔFeature = (Feature_post_ − Feature_pre_) / Feature_pre_.

### Delta-radiomics machine learning modeling

**Fig. 3:**
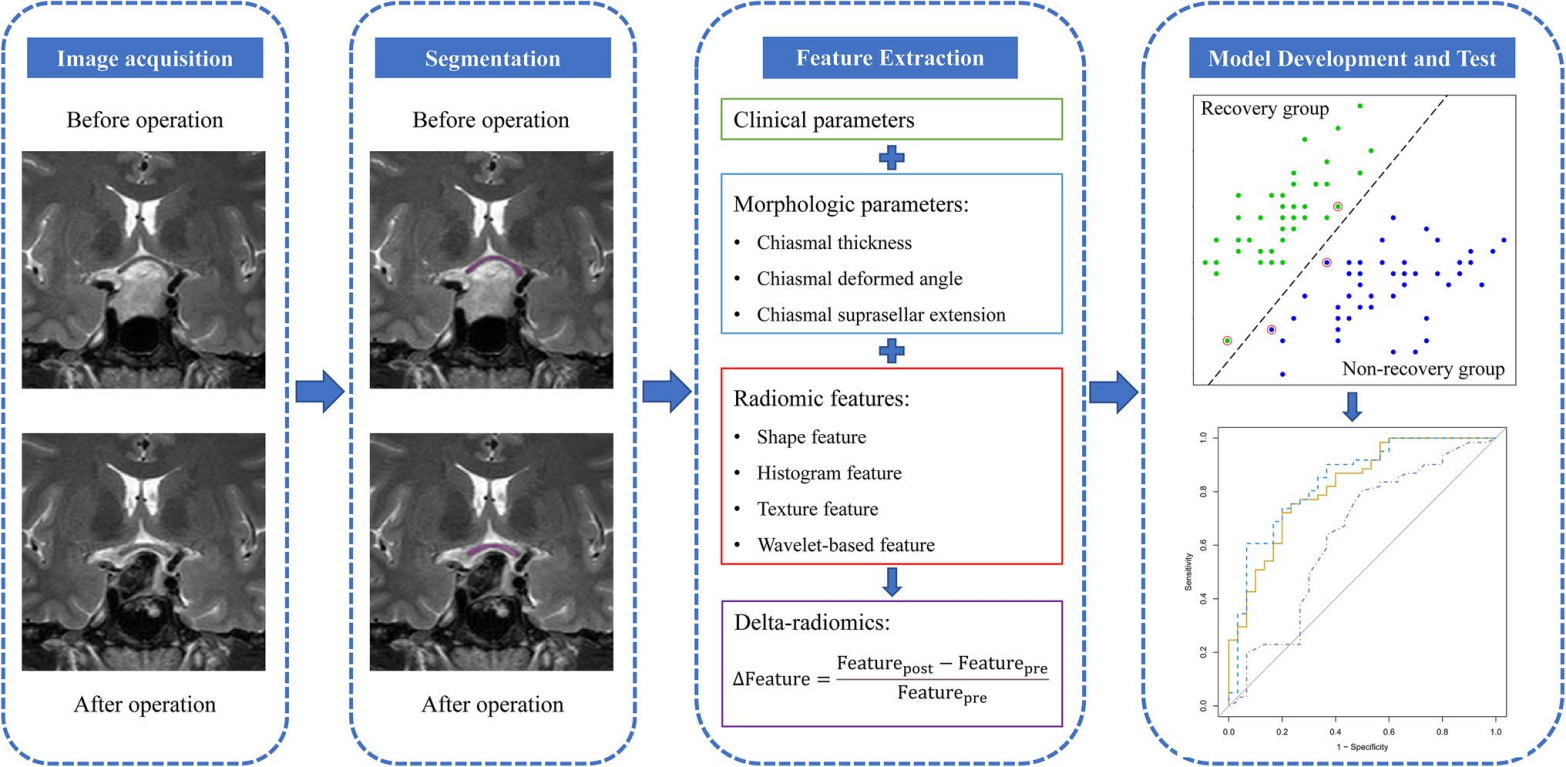
The workflow of developing machine learning models based on delta-radiomics of the optic chiasm to predict the postoperative visual outcomes of pituitary adenoma patients

The whole dataset (*n* = 130) was randomly partitioned into the development set (*n* = 91) and independent test set (*n* = 39) on the proportion of 7: 3 using stratified sampling. Our model was trained based on the delta-radiomics features in the development set by the least absolute shrinkage and selection operator (LASSO) feature selection method, wrapped with a linear support vector machine (SVM) classifier through a five-fold cross-validation approach. The model with the least overfitting and closest performance to the average performance of all five models was selected [33]. Then the model was applied in the test set which was kept untouched during the model training to obtain the generalizability of our predictive model for independent patients. The area under the receiver operating characteristic curve (AUC), F1-score, accuracy, specificity, sensitivity, positive predictive value (PPV), and negative predictive value (NPV) were used to evaluate the performance of predictive models. The calibration of the model was evaluated with calibration plots. Decision curve analysis was utilized to assess the clinical usefulness of the model [34]. All modeling algorithms were conducted using R 3.6.3. The overall workflow of this study was illustrated in Fig. 3.

### Statistical analysis

All statistical analyses were performed with IBM SPSS Statistics 21 (IBM Corp.) and R 3.6.3. Means and standard deviations are used for the representation of continuous variables, and frequencies and percentages are used for categorical variables. Analysis was carried out using t-test, Mann–Whitney U-test, and chi-square test, as appropriate. The DeLong test was used to compare AUCs of different predictive models [35]. p < 0.05 was deemed statistically significant.

## Results

### Clinical characteristics

The cohort included 130 patients (74 females, mean age: 49.2 ± 13.3 years old), of which 87 (66.9%) experienced complete VF recovery 1 year after EETS. The average age of patients in the recovery group was significantly younger than that in the non-recovery group (47.1 ± 12.2 and 53.4 ± 14.7 years, respectively; *p* = 0.010). The preoperative MD of patients in the recovery and non-recovery groups was − 7.6 ± 6.0 dB and − 9.2 ± 6.3 dB, respectively (*p* = 0.154). The maximal tumor diameter was 23.7 ± 6.9 mm in the recovery group and 24.9 ± 7.5 mm in the non-recovery group (*p* = 0.349). No significant differences were observed between the recovery and non-recovery groups in terms of gender (*p* = 0.578), histologic subtype (*p* = 0.890), and Ki-67 index (*p* = 0.061). Detailed clinical characteristics of enrolled patients were provided in Table 1. The MRI scanner distribution between the recovery and non-recovery groups was provided in Supplementary Materials 2.

**Table 1:** Clinical characteristics of patients included in this study

Characteristics	Recover group	Non-recovery group	*p* value
Case	87 (66.9%)	43 (33.1%)	
Gender			0.578
Male	36 (41.4%)	20 (46.5%)	
Female	51 (58.6%)	23 (53.5%)	
Age at diagnosis (year)	47.1 ± 12.2	53.4 ± 14.7	0.010^a^
Preoperative MD (dB)	-7.6 ± 6.0	-9.2 ± 6.3	0.154^b^
Maximal tumor diameter (mm)	23.7 ± 6.9	24.9 ± 7.5	0.349^a^
Histological subtype			0.890
Functional adenoma	15 (17.2%)	7 (16.3%)	
Non-functional adenoma	72 (82.8%)	36 (83.7%)	
Ki-67 distribution			0.061
≥ 3%	14 (16.1%)	13 (30.2%)	
< 3%	73 (83.9%)	30 (69.8%)	

*MD* mean deviation

^a^ t-test; ^b^ Mann–Whitney U-test

### Morphological parameters of the optic chiasm

**Fig. 4:**
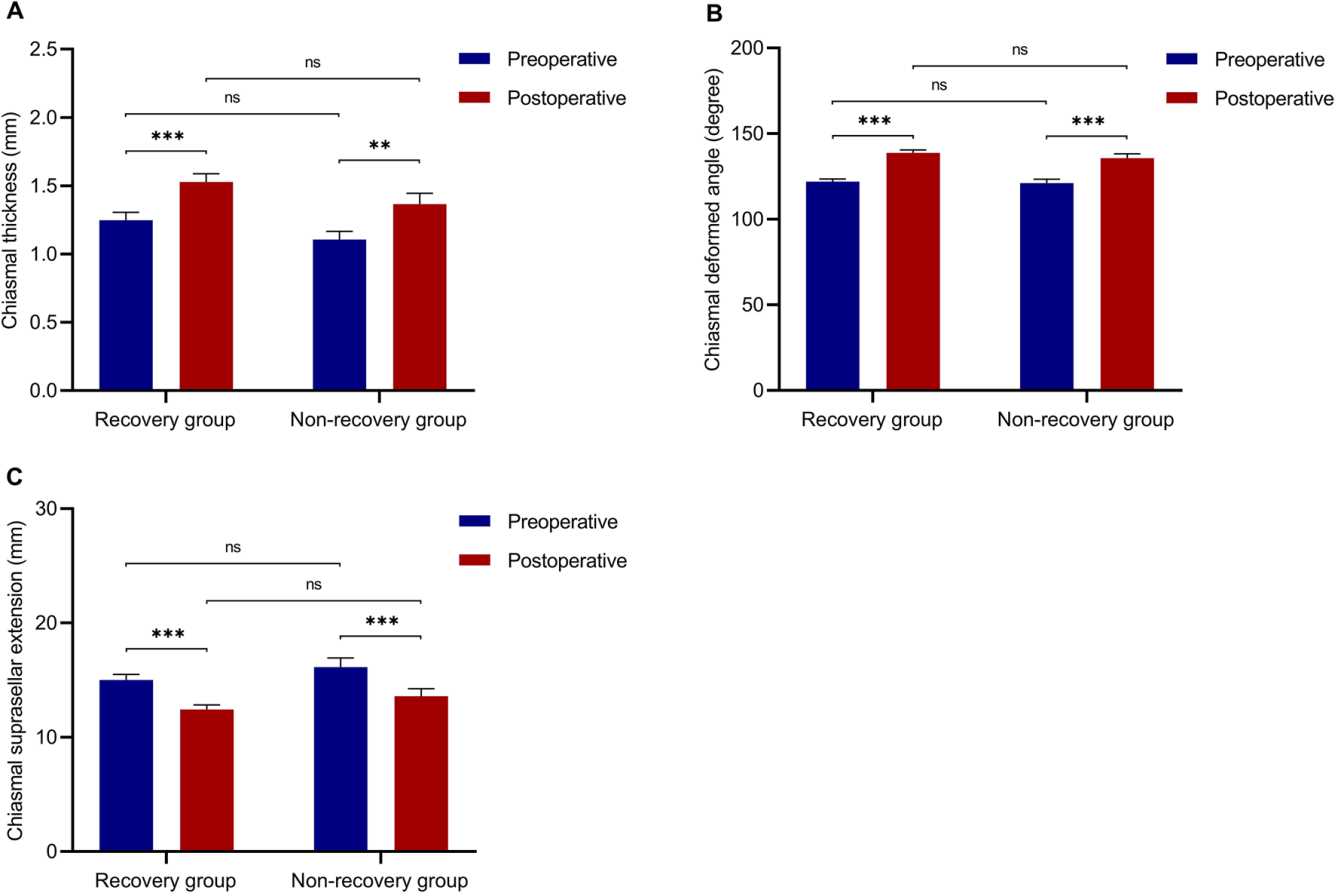
Preoperative and postoperative morphological parameters of the optic chiasm in the recovery group and non-recovery group, including chiasmal thickness (**A**), chiasmal deformed angle (**B**), and chiasmal suprasellar extension (**C**). All three morphological parameters of the optic chiasm showed significant changes after surgical decompression, while neither preoperative nor postoperative morphological parameters showed significant differences between the recovery and non-recovery group. ***: *p* < 0.001; **: *p* < 0.01; ns: not significant

There were no significant differences between the recovery group and the non-recovery group regarding preoperative or postoperative morphological parameters of the optic chiasm, including chiasmal thickness, chiasmal deformed angle, and chiasmal suprasellar extension (Fig. 4).

Compared with preoperative morphological parameters, the thickness of the optic chiasm after surgical decompression was significantly increased (*p* < 0.001), the chiasmal deformed angle was also significantly increased (*p* < 0.001) and chiasmal suprasellar extension was significantly reduced (*p* < 0.001) (Table 2). Specifically, chiasmal thickness in the recovery group and the non-recovery group was increased by 0.3 mm (40.5%) and 0.3 mm (34.2%), respectively. The chiasmal deformed angle in the recovery and non-recovery groups was increased by 16.8 degrees (14.4%) and 14.6 degrees (12.6%), respectively. Chiasmal suprasellar extension in the recovery and non-recovery groups was decreased by 2.6 mm (14.3%) and 2.5 mm (13.2%), respectively. However, neither the absolute nor relative changes in morphological parameters of the optic chiasm after surgical decompression differed significantly between the recovery group and the non-recovery group (Table 3).

**Table 2:** Comparisons between preoperative and postoperative morphological parameters of the optic chiasm

Morphological parameters	Pre-operation	Post-operation	*p* value
Chiasmal thickness (mm)	1.2 ± 0.5	1.5 ± 0.6	< 0.001
Chiasmal deformed angle (degree)	121.7 ± 14.3	137.8 ± 16.2	< 0.001
Chiasmal suprasellar extension (mm)	15.4 ± 4.8	12.8 ± 3.9	< 0.001

**Table 3:** Comparisons of preoperative, postoperative morphological parameters, and morphological changes between the recovery group and the non-recovery group

Morphological parameters	Recovery group	Non-recovery group	*p* value
Chiasmal thickness			
Preoperative value (mm)	1.2 ± 0.6	1.1 ± 0.4	0.320
Postoperative value (mm)	1.5 ± 0.6	1.4 ± 0.5	0.175
Absolute change (mm)	0.3 ± 0.7	0.3 ± 0.6	0.753
Relative change rate (%)	40.5 ± 71.1	34.2 ± 67.9	0.582
Chiasmal deformed angle			
Preoperative value (degree)	122.0 ± 13.9	121.1 ± 15.2	0.730
Postoperative value (degree)	138.8 ± 15.7	135.6 ± 17.2	0.291
Absolute change (degree)	16.8 ± 13.1	14.6 ± 12.8	0.345
Relative change rate (%)	14.4 ± 11.8	12.6 ± 12.4	0.361
Chiasmal suprasellar extension			
Preoperative value (mm)	15.0 ± 4.6	16.1 ± 5.3	0.212
Postoperative value (mm)	12.4 ± 3.7	13.6 ± 4.4	0.151
Absolute change (mm)	− 2.6 ± 3.2	− 2.5 ± 3.3	0.969
Relative change rate (%)	− 14.3 ± 20.2	− 13.2 ± 19.0	0.544

### Radiomics feature selection and evaluation

Following radiomics extraction and delta-radiomics features calculation, a total of six delta-radiomics features were selected (Supplementary Materials 3). Details regarding the feature selection process were provided in Supplementary Materials 4. The association between values of the six delta-radiomics features and the postoperative visual recovery was illustrated in Supplementary Materials 5.

Furthermore, we investigated the prognostic potentials of the presurgical values of the above six parameters. One parameter (Wavelet-LLL-GLSZM-Zone Variance) showed a significant difference between the recovery and non-recovery group and could be taken as the independent predictor (odds ratio = 1.580, *p* = 0.046) for visual recovery after correcting for the age by multivariate logistic regression analysis (Supplementary Materials 6). Receiver operating characteristic analysis showed that its AUC value was 0.653 (95% confidence interval [CI]: 0.548–0.759).

### Performance of delta-radiomics models

The predictive model for the visual recovery was constructed with the above six delta-radiomics features. With this delta-radiomics model, an AUC of 0.821 (95% CI: 0.729–0.914), with associated F1-score, sensitivity, and specificity of 0.793, 0.721, and 0.800, respectively, was obtained in the development set. This model was evaluated in the independent test set, achieving an AUC of 0.811 (95% CI: 0.692–0.930), with an F1-score of 0.889, sensitivity of 0.923, and specificity of 0.692, respectively.

**Fig. 5:**
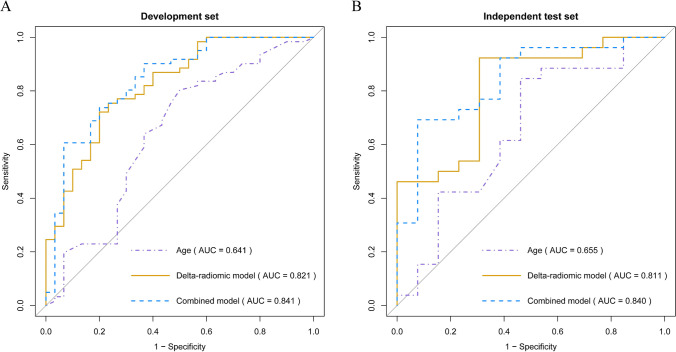
Receiver operating characteristic curves of different models in predicting the postoperative visual outcome of pituitary adenoma patients in the development set (**A**) and independent test set (**B**). The delta-radiomics model and combined model represented higher AUC than the age in both the development and independent test set

Considering age was the only clinical parameter with a significant difference between the recovery and non-recovery groups, its prognostic value for VF recovery was also investigated. AUC values of age in the development set and the independent test set were 0.641 and 0.655, respectively. The DeLong test showed that compared with the age, the delta-radiomics model represented statistically better predictive performances for the VF outcome in both the development set (*p* = 0.014) and the independent test set (*p* = 0.040). Furthermore, the combined model that incorporated age and 6 delta-radiomics features performed commendably in predicting the VF outcome, evidenced by AUC values of 0.841 (95% CI: 0.750–0.932) and 0.840 (95% CI: 0.733–0.947) in the development set and the independent test set, respectively. According to the DeLong test, predictive performances of the combined model were also statistically better than age in both the development set (*p* = 0.001) and the independent test set (*p* = 0.029). Besides, the combined model also represented comparable performance with predictors in previous studies (Supplementary Material 7). However, there were no significant differences regarding AUCs between the delta-radiomic model and the combined model in the development set (*p* = 0.364) and the independent test set (*p* = 0.497). The detailed predictive performances of different models were summarized in Table 4, and their receiver operating characteristic (ROC) curves were shown in Fig. 5.

**Table 4:** Performances of different models in predicting the postoperative visual recovery of pituitary adenoma patients in the development set and independent test set

	AUC [95% CI]	Accuracy	Sensitivity	Specificity	F1-score	PPV	NPV
Development set							
Age	0.641 [0.511—0.771]	0.703	0.803	0.500	0.784	0.766	0.556
Delta-radiomic model	0.821 [0.729—0.914]	0.747	0.721	0.800	0.793	0.880	0.585
Combined model	0.841 [0.750—0.932]	0.714	0.607	0.933	0.740	0.949	0.538
Independent test set							
Age	0.655 [0.506—0.805]	0.744	0.846	0.538	0.815	0.786	0.636
Delta-radiomic model	0.811 [0.692—0.930]	0.846	0.923	0.692	0.889	0.857	0.818
Combined model	0.840 [0.733—0.947]	0.769	0.692	0.923	0.800	0.947	0.600

*AUC* area under the receiver operating characteristic curve, *CI* confidence interval, *PPV* positive predictive value, *NPV* negative predictive value

**Fig. 6:**
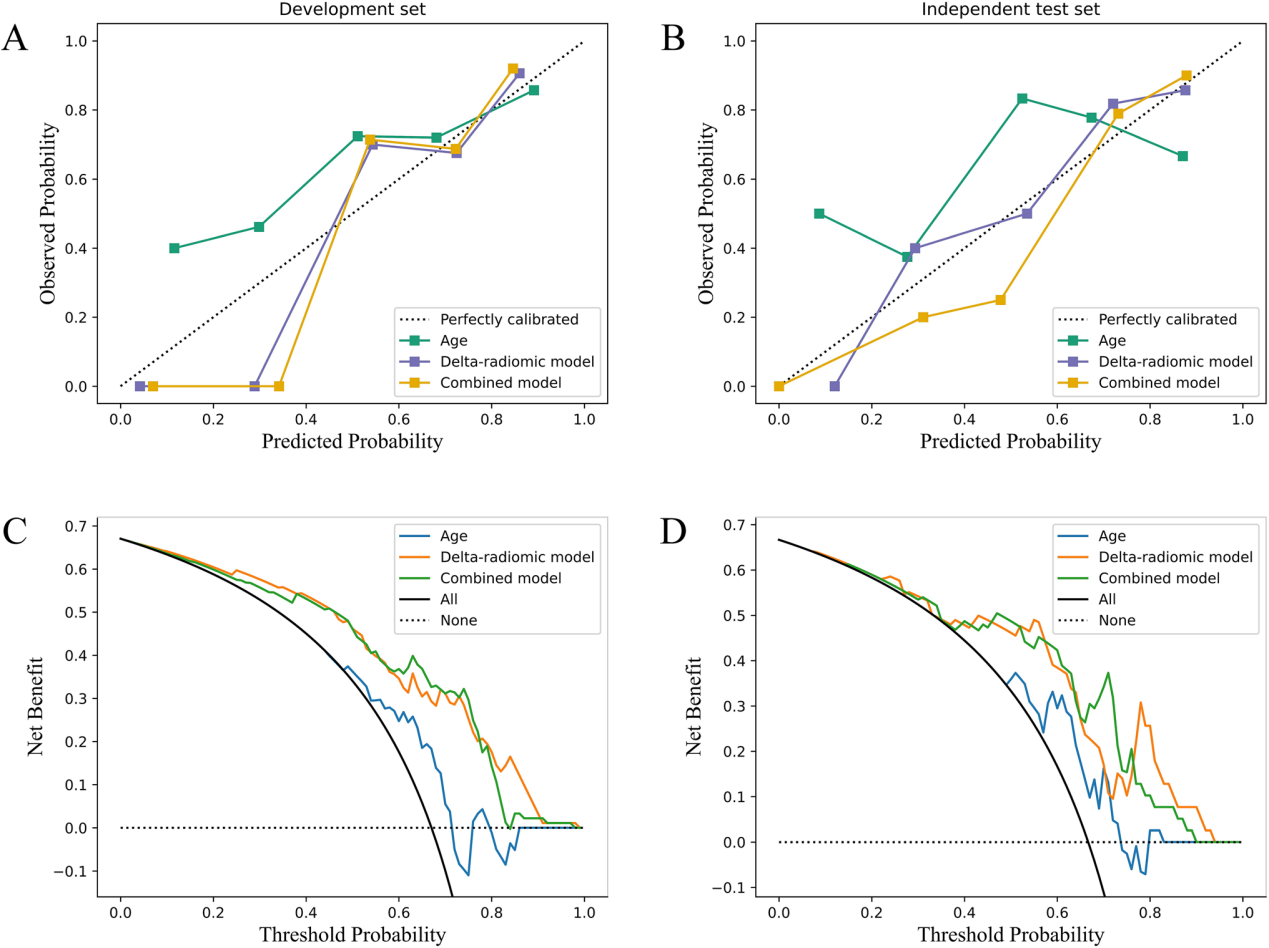
Calibration plots and decision curves of different models in the development set and independent test set. **A**, **B** The calibration plots illustrated that the delta-radiomics model and combined model showed good calibration with a closer fit to the diagonal dashed line that represents an ideal evaluation by a perfect model; **C**, **D** The decision curves illustrated that our models were clinically available. The black solid line represents all patients with visual recovery, while the black dashed line represents all patients without visual recovery

The calibration plots showed that the observed probabilities of visual recovery were consistent with the predicted probabilities of the delta-radiomics model and combined model in both the development and independent test set (Fig. 6A, B). Moreover, the decision curves illustrated that both the delta-radiomics model and combined model provided significant improvement in the net benefit compared with the age in the development and independent test set (Fig. 6C, D).

## Discussion

Despite several prognostic factors for visual outcome that have been investigated in previous research, the prediction of postoperative visual recovery for pituitary adenoma patients with a more practical and accurate approach remains a challenge [36]. Changes in morphological parameters and radiomics features of the optic chiasm on MRI after surgical decompression may reveal prognostic insights to predict the VF outcome of pituitary adenoma patients. The present study found that postoperative morphological changes of the optic chiasm could not significantly be used as predictors for the visual outcome. Instead, delta-radiomics of the optic chiasm derived from routine MR images after surgical decompression achieved feasible performances in predicting the visual outcome of pituitary adenoma patients following EETS.

The visual recovery of pituitary adenoma patients following EETS is a long-term and gradually developing process. Early visual improvement occurs immediately after surgical chiasmal decompression that leads to rapid reversal of conduction block, and the improvement of axoplasmic transport and remyelination contribute to sustained visual recovery over weeks to months after surgery [36]. Previous research demonstrated that the early morphological recovery of the optic chiasm after operation may be associated with the functional recovery of pituitary adenoma patients, and postoperative restoration of symmetry of the optic chiasm could suggest sufficient VF recovery [25]. However, the evaluation of chiasmal symmetry on MRI is relatively subjective, and quantitative analysis of the morphological recovery of optic chiasm in pituitary adenoma patients following EETS has never been reported in previous studies. Therefore, in the present study, we utilized a series of imaging parameters to quantitatively analyze early morphological changes of the optic chiasm after surgical decompression and investigated their prognostic values of VF outcome in pituitary adenoma patients. The results indicated that the thickness and deformed angle of the optic chiasm were significantly increased, and its suprasellar extension was significantly reduced within 72 h after EETS. However, these morphological changes, whether in absolute change or relative change rate, were not significantly different between the recovery group and the non-recovery group, indicating that the association between early macroscopic morphological changes of the optic chiasm after operation and functional recovery of pituitary adenoma patients may be relatively inadequate.

Compared with traditional imaging parameters, radiomics has the potential to detect the underlying pathophysiological process beyond the naked eye [18]. Given that the postoperative visual recovery of pituitary adenoma patients is a dynamic process, the delta-radiomics features of the optic chiasm that involved changes in chiasmal microscopic imaging characteristics along with surgical decompression would be more informative. In recent years, delta-radiomics has shown promising performances in predicting disease progression and monitoring treatment response [26–29]. Since previous researchers suggested the early morphological recovery of the optic chiasm after surgery was associated with long-term visual recovery, we hypothesized that delta-radiomics of the optic chiasm in the early postoperative period may predict the final visual outcome [25, 37]. Therefore, preoperative and early postoperative images were utilized to construct the predictive model. Our results showed that the machine learning model based on delta-radiomics of the optic chiasm within 72 h after surgical decompression represented good performances in predicting the VF recovery of pituitary adenoma patients, suggesting that changes of microscopic image characteristics of the optic chiasm may better reflect functional reversibility and is more relevant to the visual outcome of pituitary adenoma patients compared with macroscopic morphological changes. Six delta-radiomics features were selected as they were correlated with visual outcomes. Specifically, five parameters showed smaller changes in patients with better visual outcomes after surgery, including contrast (a measure of the spatial intensity change and the overall gray level dynamic range) from both original and wavelet images, median (the median gray level intensity within the area), and inverse variance and zone variance (a measure of the variance in the area). The possible speculation is that the more stable microscopic image patterns of the optic chiasm during surgical decompression may imply a better recovery process and visual outcome. Only the larger change of Cluster Shade (a measure of the skewness and uniformity of the gray level co-occurrence matrix) was associated with the postoperative visual recovery. Among the above six parameters, only Contrast from neighboring gray tone difference matrix has been suggested to be related to pathological changes of the optic nerve, like demyelination and axonal loss in optic neuritis patients by previous researchers [38]. Whereas, the specific relationship between the other five radiomic parameters and pathological changes in optic neuropathies has limitedly been explored. Detailed implications of these voxel-wise features to visual function and outcome require more investigations in the future.

Clinical characteristics and parameters obtained from optical coherence tomography (OCT) were commonly used to predict visual outcomes of pituitary adenoma patients with inconsistent results. In our study, increased age was a negative prognostic factor for VF recovery, which was in accordance with previous types of research [39–41]. However, the prognostic value of age is relatively limited, and both our delta-radiomics model and combined model significantly outperformed the age in predicting the VF recovery of pituitary adenoma patients. In our study, we explored the prognostic potential of preoperative morphological and radiomics parameters, although their predictive capabilities were not ideal. This could be due to the influence of the surgical procedure on visual outcomes. Combining preoperative and postoperative images may yield more accurate predictions than using only preoperative images. In addition, our combined model also represented competitive predictive performances compared with most developed models in previous studies that usually incorporated OCT-based parameters like RNFL and ganglion cell layer thickness (Supplementary Material 5) [12, 13, 30, 42–44]. One study utilized MRI compression grade to evaluate the compression degree of the optic chiasm and combined it with preoperative MD and RNFL thickness to build a predictive model for visual recovery that achieved similar performances (AUC = 0.84) to our models [12]. More importantly, compared with OCT which was not routinely conducted for pituitary adenoma patients in clinical works, our proposed models were constructed using conventional MR images that were easily obtained from routine clinical management of pituitary adenoma patients receiving EETS, indicating that our models could potentially be utilized in clinical practice to assist in predicting the visual outcome in the early postoperative period. For patients who may be predicted to have poor outcomes, timely visual rehabilitation after surgery is needed to preserve visual function as much as possible and improve quality of life.

The present study has several limitations. First, this study is limited by its single-center, retrospective nature with the inherent selection bias. Given the small sample size and wide range of 95% CI of the metrics, the real predictive performance of our delta-radiomic models may not be so optimistic. Future prospective studies are required to validate our model thoroughly in multi-institutional datasets with large cohorts of patients. Second, previously reported prognostic factors obtained from OCT were not considered in this study, as OCT was not routinely performed for pituitary adenoma patients in our center. Future research is warranted to explore whether the incorporation of OCT-based parameters with radiomics could further improve the performance of the predictive model. Third, some cases were excluded as their unrecognizable optic chiasm due to extremely severe compression by tumors. This limitation may affect the model's applicability in real-world clinical scenarios. Fourth, images within three days after the operation were used for delta-radiomics calculation based on the experiences of neurosurgeons, as they urge to predict visual outcomes in a short time after EETS. However, the visual recovery takes place for months and the assessment of recovery takes place 1 year later. More research is required to investigate the appropriate time point for the recovery prediction.

In conclusion, compared with morphological changes of the optic chiasm after surgical decompression, delta-radiomics of the optic chiasm have better prognostic values for visual recovery in pituitary adenoma patients following EETS. Based on delta-radiomics of the optic chiasm derived from routine MR images, we proposed novel machine learning models that are effective in predicting the postoperative visual outcome of pituitary adenoma patients.

### Supplementary Information

Below is the link to the electronic supplementary material.

Supplementary file1 (PDF 242 KB)

## Abbreviations

AUC: Area under the receiver operating characteristic curve
CI: Confidence interval
EETS: Endoscopic endonasal transsphenoidal surgery
IBSI: Imaging Biomarker Standardization Initiative
ICC: Intraclass correlation coefficients
LASSO: Least absolute shrinkage and selection operator
MD: Mean deviation
NPV: Negative predict value
OCT: Optical coherence tomography
PPV: Positive predict value
RNFL: Retinal nerve fiber layer
ROC: Receiver operating characteristic
ROI: Region of interests
SVM: Support vector machine
T2WI: T2-weighted imaging
VF: Visual field

## Declarations

### Guarantor

The scientific guarantor of this publication is Chaoyue Chen.

### Statistics and biometry

No complex statistical methods were necessary for this paper.

### Study subjects or cohorts overlap

No

### Methodology

- retrospective
- diagnostic or prognostic study
- performed at one institution
