# Supplementary material for: Predicting visual recovery in pituitary adenoma patients post-endoscopic endonasal transsphenoidal surgery: Harnessing delta-radiomics of the optic chiasm from MRI
Source: Eur Radiol. 2023 Jul 24;33(11):7482–93. doi: 10.1007/s00330-023-09963-9 (PMC10598191; doi:10.1007/s00330-023-09963-9)
Supplement: Supplementary file 1 — Supplementary file1 (PDF 242 KB) [file 330_2023_9963_MOESM1_ESM.pdf]

Predicting visual recovery in pituitary adenoma patients post-endoscopic endonasal transsphenoidal surgery: harnessing delta-radiomics of the optic chiasm from MRI

Electronic Supplementary Material

Supplementary Materials 1: The detailed parameters of MRI scanners in this study.

	Philips Achieva	GE Discovery MR750	Siemens Skyra
Magnetic field strength (T)	3.0	3.0	3.0
Repetition time (ms)	3000	4300	2600
Echo time (ms)	80	131	94
Slice thickness (mm)	2	2	2
Field of view (mm^2)	230×230	210×210	190×180
Voxel size (mm^3)	0.4×0.4×2.2	0.4×0.4×2.2	0.6×0.6×2.4

Supplementary Materials 2: The MRI scanner distribution between the recovery group and non-recovery group in this study.

	Development set			Independent test set		
	Recovery group (n = 61)	Non-recovery group (n = 30)	P value	Recovery group (n = 26)	Non-recovery group (n = 13)	P value
Preoperative			0.933			0.748
Philips Achieva	35 (57.4%)	16 (53.3%)		13 (50.0%)	5 (38.5%)	
GE Discovery MR750	17 (27.9%)	9 (30.0%)		8 (30.8%)	5 (38.5%)	
Siemens Skyra	9 (14.8%)	5 (16.7%)		5 (19.2%)	3 (23.1%)	
Postoperative			0.795			0.756
Philips Achieva	33 (54.1%)	14 (46.7%)		12 (46.2%)	5 (38.5%)	
GE Discovery MR750	18 (29.5%)	10 (33.3%)		9 (34.6%)	4 (30.8%)	
Siemens Skyra	10 (16.4%)	6 (20.0%)		5 (19.2%)	4 (30.8%)	

Supplementary Materials 3: The list of selected features in the delta-radiomic model.

Filter	Feature type	Feature name
Wavelet-LHH	GLCM	Inverse Variance
Original	NGTDM	Contrast
Wavelet-LLL	GLSZM	Zone Variance
Wavelet-HHL	First order	Median
Wavelet-LLL	NGTDM	Contrast
Wavelet-HHH	GLCM	Cluster Shade

GLCM, gray-level co-occurrence matrix; NGTDM, neighboring gray tone difference matrix; GLSZM, gray-level size zone matrix

Supplementary Materials 4: The flow chart of the feature selection process.

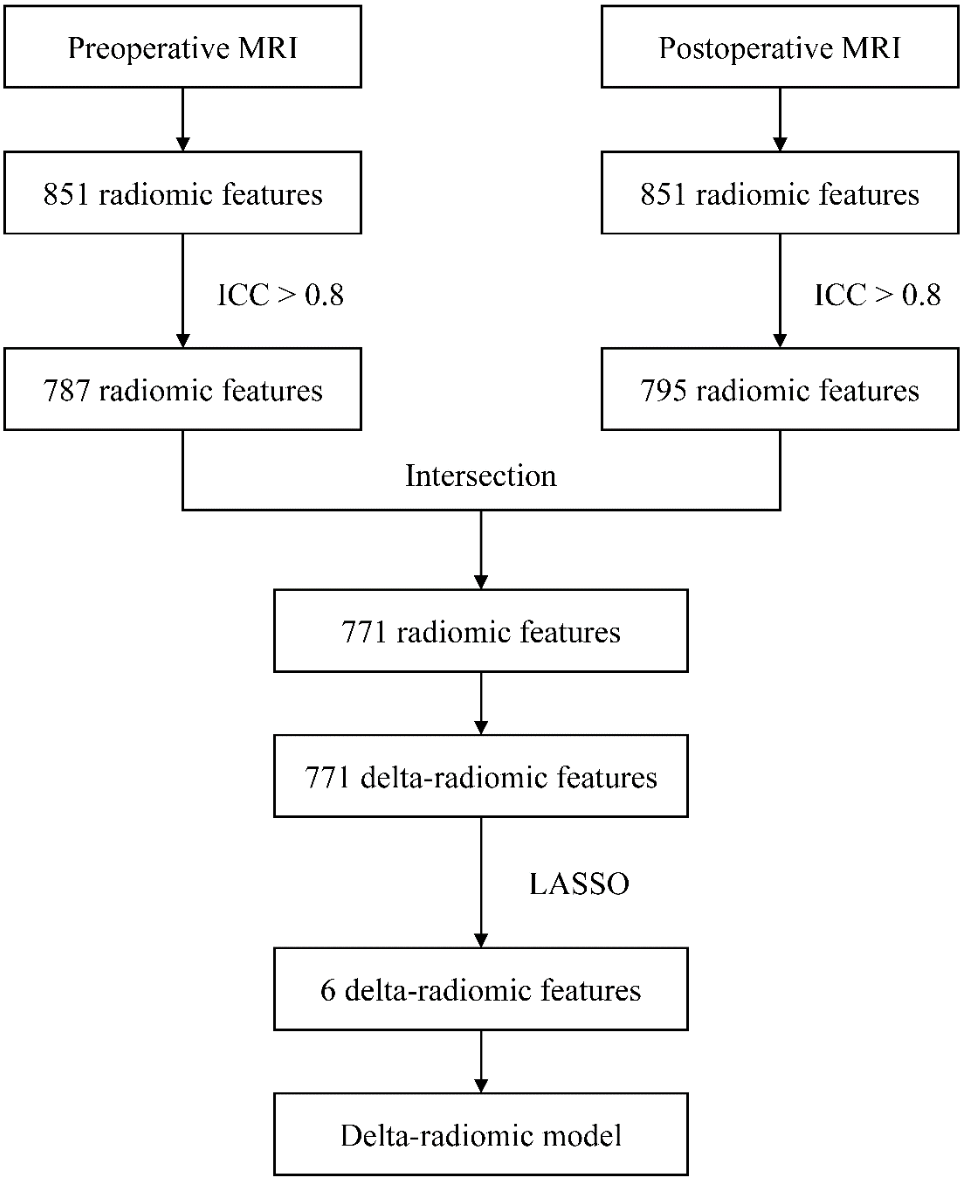

Supplementary Materials 5: The association between values of the six delta-radiomic features and the postoperative visual recovery.

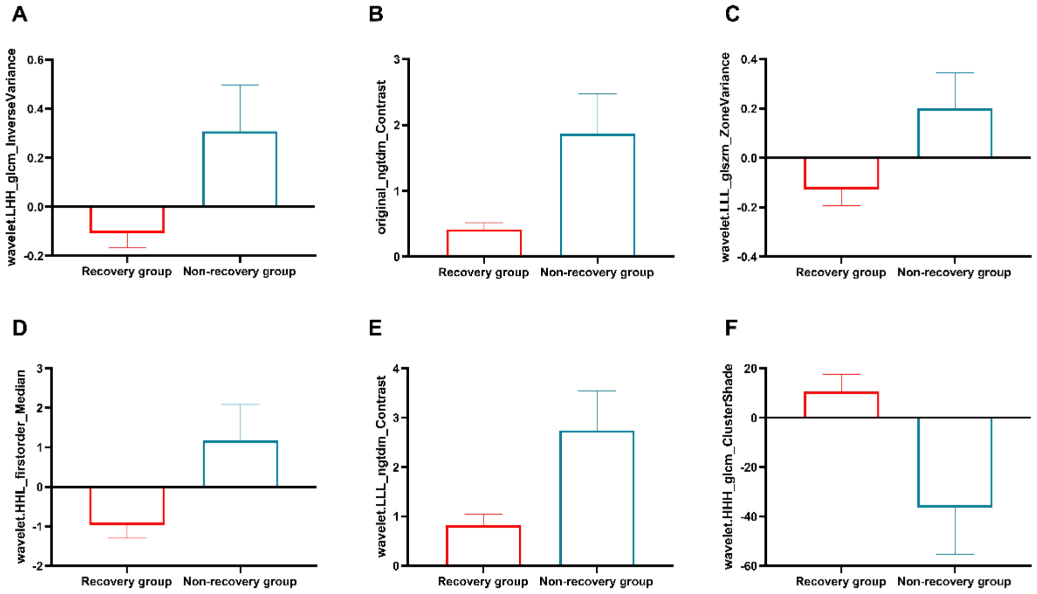

Supplementary Material 6: Results of multivariate logistic regression analysis and receiver operating characteristic analysis on presurgical radiomic parameters.

	P value	OR [95% CI]	AUC [95% CI]
Wavelet-LLL-GLSZM-Zone Variance	0.046	1.580 [1.008 - 2.476]	0.653 [0.548 - 0.759]
Age	0.026	0.632 [0.422 - 0.947]	0.647 [0.541 - 0.754]

OR, odds ratio; CI, confidence interval; AUC, area under the receiver operating characteristic curve

Supplementary Material 7: Summary of previous studies reporting predictors for postoperative visual recovery of pituitary adenoma patients.

Year	Authors	Sample size	Follow-up time	Predictors	AUC
2022	Xia, L et al	53	6 months	GCIPL thickness	0.74
2022	Meyer, J et al	108	2 years	GCL thickness	0.90
2021	Lee, G I et al	87	1 year	Combination of RNFL, GCL and IPL thickness	0.79
2020	Wang, M T M et al	239	2 years	Combination of age, MD and RNFL thickness	0.83
2020	Yoo, Y J et al	79	6 months	GCL thickness	0.96
2016	Lee, J et al	57	6 months	Combination of MRI compression grade, RNFL thickness and MD	0.84

AUC, area under the receiver operating characteristic curve; GCIPL, ganglion cell-inner plexiform layer; GCL, ganglion cell layer; RNFL, retinal nerve fiber layer; IPL, inner plexiform layer; MD, mean deviation
